# Cases of human brucellosis in Sweden linked to Middle East and Africa

**DOI:** 10.1186/s13104-016-2074-7

**Published:** 2016-05-17

**Authors:** Giuliano Garofolo, Antonio Fasanella, Elisabetta Di Giannatale, Ilenia Platone, Lorena Sacchini, Tiziana Persiani, Talar Boskani, Kristina Rizzardi, Tara Wahab

**Affiliations:** National and OIE Reference Laboratory for Brucellosis, Istituto Zooprofilattico Sperimentale dell’Abruzzo e del Molise “G. Caporale”, Teramo, Italy; Istituto Zooprofilattico Sperimentale della Puglia e della Basilicata, Foggia, Italy; Department of Microbiology, Public Health Agency of Sweden, Stockholm, Sweden

**Keywords:** Brucellosis, *Brucella melitensis*, MLVA

## Abstract

**Background:**

Human brucellosis cases are still reported each year in Sweden despite eradication of the disease in animals. Epidemiological investigation has never been conducted to trace back the source of human infection in the country. The purpose of the study was to identify the source of infection for 16 human brucellosis cases that occurred in Sweden, during the period 2008–2012.

**Results:**

The isolates were identified as *Brucella melitensis* and MLVA-16 genotyping revealed 14 different genotypes of East Mediterranean and Africa lineages. We also reported one case of laboratory-acquired brucellosis (LAB) that was shown to be epidemiological linked to one of the cases in the current study.

**Conclusions:**

*Brucella melitensis* was the only species diagnosed, confirming its highest zoonotic potential in the genus *Brucella*, and MLVA-16 results demonstrated that the cases of brucellosis in Sweden herein investigated, are imported and linked to travel in the Middle East and Africa. Due to its zoonotic concerns, any acute febrile illness linked to recent travel within those regions should be investigated for brucellosis and samples should be processed according to biosafety level 3 regulations.

**Electronic supplementary material:**

The online version of this article (doi:10.1186/s13104-016-2074-7) contains supplementary material, which is available to authorized users.

## Background

Brucellosis is one of the most reported zoonosis worldwide with an emergence of new foci of both human and animal disease related to socio economic changes [1]. Human-human transmission has occasionally been reported [2]. Transmission mainly occurs through the ingestion of contaminated raw milk and dairy products, via professional exposure, or via accidental inhalation of the *Brucella* culture [3, 4]. In human beings the disease is a septicemic febrile illness frequently associated with localized bone and tissue infections [5]. In animals it causes abortion and fertility problems resulting in considerable financial losses [6]. Brucellosis occurs widely throughout the world, particularly in developing countries where small ruminants are farmed. The diagnosis of *Brucella* is a challenge, and in low risk countries, the gold standard method used is the microbiological isolation from clinical samples. However, due to its pathogenicity, a biosafety level 3 (BSL-3) laboratory must be used to handle potentially positive samples. *Brucella* infection is one of the major common laboratory-acquired infections in the United States [7]. In these cases inhalation of infective aerosol is the most common route of transmission, but direct contact with clinical specimens should not be ignored [8]. Brucellosis is a rare infection in humans in the EU. The highest notification rates and the majority of the autochthonous cases were reported from Mediterranean countries. Brucellosis in livestock has been eradicated in Sweden, but a number of human cases are registered annually from individuals that have travelled in brucellosis risk zones [9]. Commonly in Sweden, the brucellosis cases are found among travellers returning from endemic countries. Nevertheless, the febrile cases are rarely investigated for brucellosis in the absence of suspected anamnesis. This is the first study in Sweden in which brucellosis is studied in relation to the geographic source. We confirmed the diagnosis and we identified the source of infection for 16 cases of brucellosis detected over a four-year period (2008–2012) by means of PCR typing and MLVA genotyping. Here we applied MLVA in order to understand the geographic origin of the cases, and if there were clusters of infection as a result of food poisoning. Our findings revealed that all 16 cases of human brucellosis in Sweden studied were caused by *B. melitensis* lineages originating from the Middle East and Africa. A paradigmatic case of laboratory-acquired brucellosis (LAB) is also described pointing out the necessity of raising awareness in Sweden for contagious risk in laboratory staff.

## Results

All isolates were initially cultured on sheep blood agar and DNA was prepared using the commercially available EZ1 DNA Tissue kit (Qiagen, Stockholm, Sweden). The *AbortusMelitensisOvisSuis* (AMOS) polymerase chain reaction (PCR) was used for identifying the *Brucella* species, prior to multiplex PCR [10, 11]. Then isolates were genotyped using the MLVA-16 panel of Le Flèche et al. [12] with modifications as described by Al Dahouk et al. [13]. We used the MLVA-16 protocol that uses multiplex PCRs and multicolour capillary electrophoresis [14], [15]. The MLVA data of the 16 strains were compared to genotypes from isolates available in the MLVA bank on the website (http://mlva.u-psud.fr/). Phylogenetic and cluster analysis was performed using the unweighted pair group method with arithmetic mean (UPGMA) in PAUP* 4.0b [16] using the allele data analyzed as alpha codes, aggregating the MLVA data from this study with MLVA data from the East Mediterranean and Africa lineages.

**Fig. 1:**
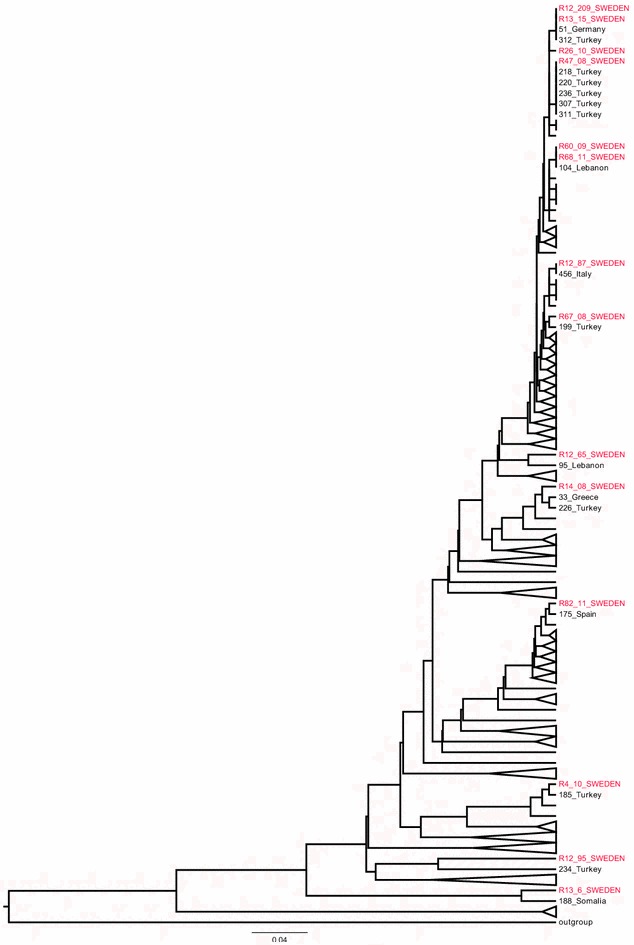
UPGMA assessment of relationships MLVA-16 profiles for 16 *B. melitensis* isolates from Sweden and 455 *B. melitensis* of West Mediterranean and Africa lineages available from MLVA bank. Swedish isolates are highlighted in *red*, other strains are identified by progressive numbers and geographic source. The branches unrelated are collapsed

AMOS PCR assigned all the 16 strains to the *B. melitensis* species, which is highly pathogenic to humans. MLVA genotyping identified 14 different genotypes designated with the letters from *A* to *N* (Table 1, Additional file 1) [13, 17, 18]. The *B. melitensis* R13_15 strain was cultured from a sample provided by a laboratory trainee (a 38-year-old female) after she began experiencing nonspecific symptoms of malaise. Three months previously she had handled a suspicious sample (later identified as strain R12_209) in a BSL-2 laboratory. No evidence of exposure to brucellosis, other than through occupational exposure, was identified. The epidemiological investigation showed that the trainee performed gram staining while other laboratory staff performed phenotypical tests, but at that time all of them were unaware that they were dealing with *Brucella*. The culture was then sent to the Public Health Agency of Sweden, where *Brucella* was readily identified by culture, real time PCR, and matrix-assisted laser desorption-ionization time-of-flight (MALDI-TOF). The R12_209 strain was isolated from a 65-year-old man with a febrile illness after returning to Sweden from Iraq in October 2012. MLVA linked the strains R13_15 and R12_209 showing a similar genotype (Table 1). Two additional strains R60_09, R68_11 also had similar genotypes and were isolated from patients moved from Kurdistan (Iraq) but in different years without any apparent connection. Interestingly five out of fourteen MLVA-16 genotypes (*A, C, J, K*, and *N*) were previously identified from other authors in patients with recent history of travelling from brucellosis high risk areas. Moreover MLVA searching in the cooperative database of the MLVA bank revealed that the *B. melitensis* R13_6 strain, isolated from a patient moved from Somalia, belonged to the Africa lineage, while the remaining *B. melitensis* strains showed the East Mediterranean lineage for the patients with nationalities from different Middle East countries. The UPGMA clustering method revealed that all the isolates were diverse and widely distributed throughout the *B. melitensis* phylogeny (Fig. 1). The findings demonstrated a multi foci of infection with potentially different geographic sources.

**Table 1:** *Brucella melitensis* isolates studied

Strain id	Year	Patient nationality	Species	Lineage	MLVA8^a^	MLVA11^b^	MLVA16	Comments
R12_209	2012	Iraqi	*B. melitensis*	East Mediterranean	43	125	A	Exact match with human strains from Germany as introduction from Turkey [13], and from Turkey [18]
R12_65	2012	Iraqi	*B. melitensis*	East Mediterranean	43	Novel	B	
R12_87	2012	Iraqi	*B. melitensis*	East Mediterranean	43	125	C	Exact match with human strain from Italy as introduction from Syria [14]
R12_95	2012	Iraqi	*B. melitensis*	East Mediterranean	Novel	Novel	D	
R13_15	2013	Swedish	*B. melitensis*	East Mediterranean	43	125	A	Exact match with human strains from Germany as introduction from Turkey [13], and from Turkey [18]
R13_6	2013	Somali	*B. melitensis*	Africa	94	178	E	
R14_08	2008	Iraqi	*B. melitensis*	East Mediterranean	43	106	F	
R15_08	2008	Syrian	*B. melitensis*	East Mediterranean	43	125	G	
R26_10	2010	Iraqi	*B. melitensis*	East Mediterranean	43	125	H	
R4_10	2010	Afghan	*B. melitensis*	East Mediterranean	63	111	I	
R47_08	2008	Iraqi	*B. melitensis*	East Mediterranean	43	125	J	Exact match with human strains from Turkey [18]
R60_09	2009	Kurds	*B. melitensis*	East Mediterranean	43	125	K	Exact match with human from Lebanon [17]
R67_08	2008	Kurds	*B. melitensis*	East Mediterranean	43	125	L	
R68_11	2011	Kurds	*B. melitensis*	East Mediterranean	43	125	K	Exact match with human from Lebanon [17]
R82_11	2011	Iraqi	*B. melitensis*	East Mediterranean	42	116	M	
R95_11	2011	Kurds	*B. melitensis*	East Mediterranean	43	125	N	Exact match with human strains from Turkey [13, 18]

MLVA profiles are provided as Additional file 1

^a, b^ MLVA8 and MLVA11 genotypes are designed according to the *Brucella* cooperative database of the MLVA bank, while MLVA16 genotypes are herein assigned with capital letters (A–N)

In this study we confirm that MLVA continues to demonstrate its effectiveness as a molecular epidemiological tool of bacterial pathogens such as *Brucella* [15]. The specific level of discrimination offered by VNTRs methodology has put them as gold standard markers in the field of molecular epidemiology. MLVA typing is therefore useful to understand the epidemiological context where the cases are occurring. MLVA has been used to confirm the source of a LAB [19] as well as to demonstrate the endemicity or distinct clusters of disease [15]. The genetic fingerprints found demonstrate that the 16 *B. melitensis* isolates reported in this study are linked to Middle East and African areas, in agreement with a previous publication [20]. Unfortunately, case information is not available for our isolates, but recent immigration or travel from countries with endemic brucellosis might be a common feature for all patients as corroborated by patient nationalities (Table 1). The genetic lineages discovered put the root of the infections in the Middle East and Africa and this reflects the Swedish migration trends of groups from Iraq, Afghanistan and Somalia (source: Statistics Sweden).

Our data demonstrates that in Sweden, human brucellosis may be linked to people with a characteristic anamnesis with recent migration from brucellosis endemic areas. The majority of cases in northern Europe are travel associated. In addition, European countries may also experience domestically acquired cases. These can occur in immigrants from endemic areas or be due to private import of unpasteurized dairy products from endemic areas. Food-borne brucellosis would act as a point cluster disease but the high genetic diversity found would exclude the presence of such food poisoning clusters in our cases.

A laboratory-acquired infection was also traced back by MLVA confirming the suspected epidemiological link between the lab trainee and the brucellosis case. BSL-2 laboratory practices can potentially lead to *Brucella* infections.

It is therefore extremely important to put brucellosis in the differential diagnosis of any acute febrile illness, especially in individuals who have recently moved or travelled from such areas. In those cases, microbiologists working with suspected cases of *Brucella* must follow BSL-3 standards microbiological safety procedures to minimize the risk of infection.

## Additional file

10.1186/s13104-016-2074-7 MLVA profiles for the *B. melitensis* analyzed in this study.

## Abbreviations

PCR: polymerase chain reaction
SPP: species
VNTR: variable number tandem repeat
MLVA: multi locus VNTRs analysis
BSL: biosafety level
AMOS: *Abortus Melitensis Ovis Suis*
MALDI-TOF: matrix-assisted laser desorption-ionization time-of-flight
UPGMA: unweighted pair group method with arithmetic mean
LAB: laboratory-acquired brucellosis
